# Anti-alcohol abuse drug disulfiram inhibits human PHGDH via disruption of its active tetrameric form through a specific cysteine oxidation

**DOI:** 10.1038/s41598-019-41187-0

**Published:** 2019-03-18

**Authors:** Quentin Spillier, Didier Vertommen, Séverine Ravez, Romain Marteau, Quentin Thémans, Cyril Corbet, Olivier Feron, Johan Wouters, Raphaël Frédérick

**Affiliations:** 1 Medicinal Chemistry Research Group (CMFA), Louvain Drug Research Institute (LDRI), Université Catholique de Louvain, B-1200 Brussels, Belgium; 2 Pole of Pharmacology and Therapeutics (FATH), Institut de Recherche Expérimentale et Clinique (IREC), Université Catholique de Louvain, B-1200 Brussels, Belgium; 3 de Duve Institute, Université catholique de Louvain, B-1200 Brussels, Belgium; 4 UMR-S1172 - JPArc - Centre de Recherche Jean-Pierre AUBERT Neurosciences et Cancer, Université de Lille, Inserm, CHU Lille, F-59000 Lille, France; 5 Department of Chemistry, NAmur MEdicine & Drug Innovation Center (NAMEDIC-NARILIS), Université de Namur, 61 rue de Bruxelles, B-5000 Namur, Belgium

## Abstract

Due to rising costs and the difficulty to identify new targets, drug repurposing appears as a viable strategy for the development of new anti-cancer treatments. Although the interest of disulfiram (DSF), an anti-alcohol drug, to treat cancer was reported for many years, it is only very recently that one anticancer mechanism-of-action was highlighted. This would involve the inhibition of the p97 segregase adaptor NPL4, which is essential for the turnover of proteins involved in multiple regulatory and stress-response intracellular pathways. However, recently DSF was also reported as one of the first phosphoglycerate dehydrogenase (PHGDH) inhibitors, a tetrameric enzyme catalyzing the initial step of the serine synthetic pathway that is highly expressed in numerous cancer types. Here, we investigated the structure-activity relationships (SAR) of PHGDH inhibition by disulfiram analogues as well as the mechanism of action of DSF on PHGDH via enzymatic and cell-based evaluation, mass spectrometric and mutagenesis experiments.

## Introduction

Disulfiram (bis(diethylthiocarbamoyl) disulfide = DSF), commercially known as Antabuse, is used since 1948 (FDA-approved in 1951) as an alcohol-aversive agent for the treatment of alcohol dependence^1^. Its mechanism of action probably involves an increase of the body’s sensitivity to ethanol by inhibition of the enzyme acetaldehyde dehydrogenase (ALDH)^2^.

Starting from the 2000s, numerous studies have reported anti-tumoral properties for DSF^3,4^ and its repurposing in the therapy of cancer is foreseen. This would provide a new effective drug, avoiding expensive development phases before its commercialization^5,6^, DSF having a well-controlled ADME profile^7^ and a fairly broad efficiency on various tumor lines in pre-clinical models^8^.

Different mechanisms accounting for the anticancer activity of DSF were suggested. The group of Cassidy showed for instance in 2003 that DSF was able to inhibit nuclear factor-kappa B (NF-κB), a protein implicated in immune response, hence preventing the resistance of cancer cells to 5-fluorouracil (5-FU)^9^. Other data evidenced that DSF was able to induce apoptotic cell death of breast cancer cell lines by inhibition of the proteasomal machinery^3^. However it is only very recently that a clear anticancer mechanism for DSF was detailed when Skrott *et al*. demonstrated that an *in vivo* metabolite of DSF could act as an inhibitor of NPL4, an adaptor of segregase p97 (also called VCP), essential for the recycling of proteins involved in multiple regulatory and stress-response intracellular pathways^10^. In fact, in the body, DSF is metabolized to ditiocarb (diethyldithiocarbamate, DTC) and other metabolites. It is also known that DSF chelates bivalent metals and forms complexes with copper (Cu), which enhances its anti-tumour activity. The group of Bartek actually demonstrated that a DTC–copper complex named *bis*(diethyldithiocarbamate)–copper (CuET) forms *in vivo*, thereby providing the anti-cancer metabolite of DSF^10^.

Finally, in 2016^11^, the Cantley lab demonstrated that DSF was also a potent inhibitor of phosphoglycerate dehydrogenase (PHGDH), the first and limiting step of the so-called serine synthetic pathway (SSP)^12^, thus suggesting that DSF itself could display anticancer properties *via* an alternative mechanism-of-action. In fact, in 2011, the group of Possemato *et al*. demonstrated that PHGDH silencing leads to a significant decrease in tumor proliferation in several PHGDH-overexpressing cells^13^. However, tumorigenesis supported by PHGDH still need to be detailed.

Recent results in our lab led to the identification of new PHGDH inhibitors following a drug screening campaign^14^ and, similarly to the Cantley lab, DSF was identified with an IC_50_ of 0.59 µM. Given the importance of PHGDH in cancer metabolism and the growing interest in repurposing DSF for cancer therapy, we set out to examine structure–activity relationships (SAR) in a series of DSF analogs and to elucidate its mechanism-of-action.

## Results and Discussion

As a first step to detail our understanding of the binding of DSF on PHGDH, some preliminary SAR were investigated around the bis(dithiocarbamate) central core. To this end, a library of 20 DSF analogues developed earlier^15^ by our team was screened on purified PHGDH using an isolated enzymes inhibition assay^14^. Compared to the parent compound (DSF = **1**), apart from compound **2**, all the attempts to replace the diethylamino side chain in DSF (**3**–**16**) afforded similarly active compounds (Table 1). On the contrary, replacing the central symmetric bis(dithiocarbamate) motif by either an acetamido carbamodithioate (**17**), a thioacetamido carbamodithioate (**18**), or a methylene dicarbamidodithioate (**19–20**) led to compounds that inhibit PHGDH only weakly or inactive compounds. These relatively flat SAR for DSF analogues possessing the symmetrical bis(dithiocarbamate) central core (**1,3–16**) led us to suspect either (i) unspecific binding, (ii) binding at a site distant from the PHGDH active site (allosteric binding) or possibly (iii) covalent inhibition. Puzzled by this question we set out to detail the mechanism-of-action of DSF on PHGDH.

**Table 1:** PHGDH inhibition (IC_50_) of DSF analogues **1–20**.


Cmpd	R	PHGDH inhibition (IC_50_, µM)^a^	Cmpd	R	PHGDH inhibition (IC_50_, µM)^a^
**1 (DSF)**		0.59	**11**		0.41
		[0.37–0.95]			[0.27–0.60]
**2**		>1 mM	**12**		22.5
					[14.0–39.9]
**3**		1.39	**13**		0.38
		[1.03–1.89]			[0.29–0.50]
**4**		0.51	**14**		0.36
		[0.45–0.77]			[0.20–0.62]
**5**		0.58	**15**		0.42
		[0.44–0.77]			[0.33–0.54]
**6**		0.42	**16**		1.01
		[0.36–0.48]			[0.67–3.52]
**7**		0.21	**17**		>1 mM
		[0.18–0.25]			
**8**		0.34	**18**		36.38
		[0.23–0.51]			[19.99–66.19]
**9**		0.17	**19**		45.86
		[0.14–0.21]			[29.15–72.16]
**10**		0.57	**20**		67.32
		[0.46–0.69]			[29.42–154.18]

^a^All experiments to determine IC_50_ values were performed in triplicates at each compound dilution. Under bracket: 95% confidence interval.

Unspecific binding was ruled out by adding, in the inhibition assay buffer, Triton-X, a detergent well-known to abrogate inhibition data *via* unspecific binding, and measuring a similar IC_50_ (See Supporting Information Fig. S1).

Then, both a rapid dilution and an incubation assays were performed to investigate the possible formation of a covalent adduct between PHGDH and DSF as already suggested on other targets^16^.

**Figure 1:**
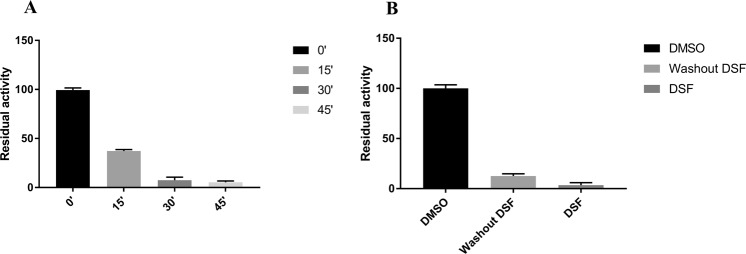
Characterization of PHGDH inhibition by DSF. Residual activity percentage of PHGDH (**A**) upon incubation with DSF (50 µM) for the indicated times and (**B**) after the rapid dilution assay experiment with DSF (50 µM). All experiments values were performed in triplicates at each compound dilution and error bars show the standard deviation. Data were collected at 37 °C with a PHGDH concentration of 12 ng/µL in 50 mM Tris and 1 mM EDTA at pH 8.5.

As reported on Fig. 1A, PHGDH inhibition increases, along incubation time, from no inhibition (100% residual activity) in the absence of DSF, to 100% inhibition after 45 min incubation with DSF. These results suggest that DSF acts as a time-dependent inhibitor on PHGDH. Moreover, after a rapid dilution of the enzyme/inhibitor complex, the PHGDH activity was not restored indicating that DSF shows most probably an irreversible inhibition mechanism (Fig. 1B).

**Figure 2:**
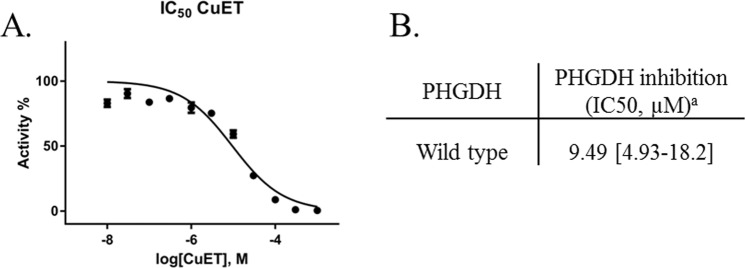
(**A**) Dose-response curve of CuET on WT PHGDH. (**B**) PHGDH inhibition (IC_50_) of CuET^a^. All experiments to determine IC_50_ values were performed in triplicates at each compound dilution.Under bracket: 95% confidence interval. Data were collected at 37 °C with a PHGDH concentration of 12 ng/µL in 50 mM Tris and 1 mM EDTA at pH 8.5.

Because previous studies showed that DSF anti-cancer activity is copper-dependent, and Skrott *et al*. found that the DSF metabolite diethyldithiocarbamate containing copper ions (CuET) is responsible for the anti-cancer activity through inhibiting the p97 segregase adaptor NPL4, we verified whether PHGDH inhibition could result from the formation of this copper complex. To this end, we synthesized the diethyldithiocarbamate (DTC)-copper complex CuEt and analyzed wtPHGDH inhibition. As a result, an IC_50_ in the 10 µM range, that is about 16-fold weaker compared to DSF itself, was obtained (Fig. 2). These data demonstrate that although CuEt is known to be responsible, at least in part, for the anticancer activity of DSF, PHGDH inhibition is not driven by the formation of this DSF metabolite copper complex.

**Figure 3:**
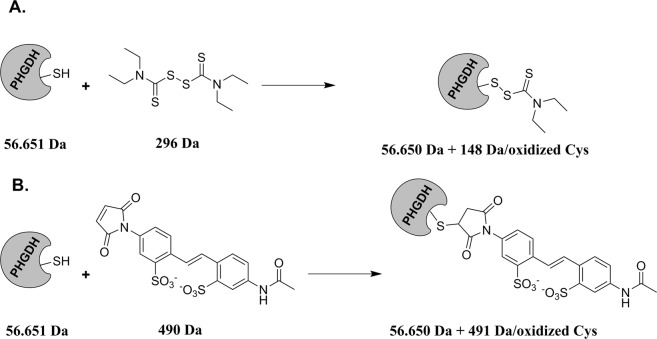
Proposed Mechanism of Interaction between PHGDH and (**A)** Disulfiram (**B)** 4-acetamido-4′-maleimidylstilbene-2,2′-disulfonic acid (**DSF** and **AMS** reacts with sulfhydryl groups of free (reduced) cysteine residues forming a mixed disulfide). Masses after coupling are given for only one reactive cysteine.

**Figure 4:**
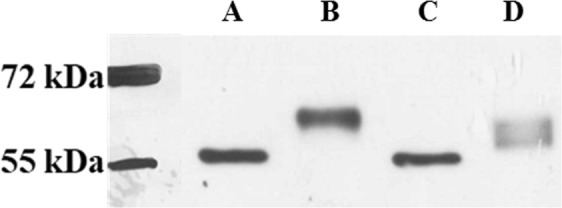
Western-blot of the different conditions. All samples were incubated with DSF and/or AMS during 1 h at room temperature before running on a 12% SDS-Tris-Glycine Page gel. **A** PHGDH. **B** PHGDH with 2 mM AMS. **C** PHGDH with 100 µM DSF. **D** PHGDH with 100 µM DSF and 2 mM AMS. Original uncropped Western-blot is available in the Supplementary Information File Fig. S3.

Next, because DSF is known to oxidize cysteine residues through the formation of a disulfide bridge such as depicted in Fig. 3A, we incubated PHGDH with 100 μM of disulfiram for 2 h and performed a western blotting of PHGDH alone and after incubation with DSF. As a control we also used a reference thiol-modification assay, the 4-acetamido-4′-maleimidylstilbene-2,2′-disulfonic acid (AMS)^17^. This thiol-modifying agent is known to bind to free sulfhydryl group of cysteine, providing a 462 Da size shift in protein mass for each cysteine oxidized thus resulting in a change in migration (Fig. 3), a phenomenon similar to what we expect with DSF although with a mass shift of 147 Da by cysteine potentially oxidized. As observed from Fig. 4, when comparing PHGDH alone (line A) and PHGDH after incubation with DSF (line C) no clear shift in mass between the two lines is observed, probably reflecting a very small shift in mass by the reaction of DSF at a specific cysteine residue. On the contrary, a clear and large shift in mass can be seen when comparing PHGDH alone (line A) with PHGDH after incubation with AMS (line B), indicating the oxidation of several cysteine residues by AMS. Finally, when PHGDH is first incubated with DSF and then with AMS a broader mass profile is observed, suggesting, in part, a competition between AMS and DSF leading to several distinct masses (line D). Altogether these results support the hypothesis that PHGDH is oxidized by DSF through cysteine(s) modification.

To unambiguously confirm this hypothesis and possibly identify the cysteine residues involved in this interaction, mass spectrometry experiments were undertaken. Briefly, PHGDH was incubated for 30 min with various concentrations of disulfiram (0.1x IC_50_, 1x IC_50_ and 10x IC_50_). At the end of the incubation, chloroacetamide was added and incubated 15′ in large excess in order to block the remaining free cysteines residues for the following analysis. The protein was separated from the medium (removal of the remaining chloroacetamide and DSF by liquid/liquid extraction with CHCl_3_/H_2_O) followed by trypsinization and analysis of the peptides by nanoUHPLC/MS.

**Figure 5:**
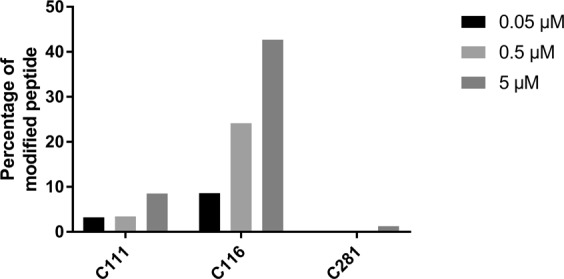
Percentage of the three oxidized cysteine residues (C111, C116 and C281) at tested concentrations. Determined from the peptide spectrum matches (PSMs) of their precursors after trypsinization and analysis by nanoUHPLC/MS. DSF at various concentrations was incubated with PHGDH in 50 mM Tris and 1 mM EDTA at pH 8.5.

The results indicated that peptides including 12 out of the 13 Cys residues of the full length PHGDH could be identified and tracked, to allow a semiquantitative determination of their relative abundances before and after DSF treatment based on the total number of identified peptides sequences. From these 12 cysteine residues, 9 are not oxidized upon DSF treatment and 3 are subject to oxidation (Cys111, 116, 281), although at very different levels (See Supporting Information Table S1). Among these 3 cysteine residues, only two, Cys116 and to a lesser extent Cys111, are found to be oxidized by DSF at the IC_50_ concentration that is 0.5 µM (Fig. 5). Interestingly, these data are in agreement with recent results from the team of Marletta^18^ which demonstrated that PHGDH could also be inhibited by the specific S-nitrosation of the same Cys116 residue. This particular cysteine thus seems to play a crucial role in PHGDH activity and moreover could constitute a novel interaction site for PHGDH inhibition.

Altogether, these data strongly suggest a mechanism of action for DSF on PHGDH involving oxidation of the Cys116 residue. To validate this hypothesis, we set out to investigate the inhibitory potency of DSF on a mutant form of PHGDH where the Cys116 was mutated to a serine residue (PHGDH C116S). Interestingly, this mutant is known to retain a catalytic activity comparable to the wild type enzyme^18^. We actually showed that the human PHGDH C116S mutant is only weakly inhibited by DSF, with a 20-fold decrease in the inhibitory potency, in comparison to the wild type enzyme (Table 2). Although this observation is a clear indication that C116 oxidation is critical for PHGDH inhibition, it also suggests that oxidation of other cysteine’s such as C111 and C281 might be involved in PHGDH inhibition albeit to a lesser extent as demonstrated recently in the works of Marletta^18^.

**Table 2:** WT and C116S PHGDH inhibition (IC_50_) of DSF.

PHGDH	PHGDH inhibition (IC_50_, µM)^a^
Wild Type	0.59 [0.37–0.95]
C116S mutant	10.23 [6.68–15.65]

^a^All experiments to determine IC_50_ values were performed in triplicates at each compound dilution, and all IC_50_ values were averaged when determined in two or more independent experiments. Under bracket: 95% confidence interval.

**Figure 6:**
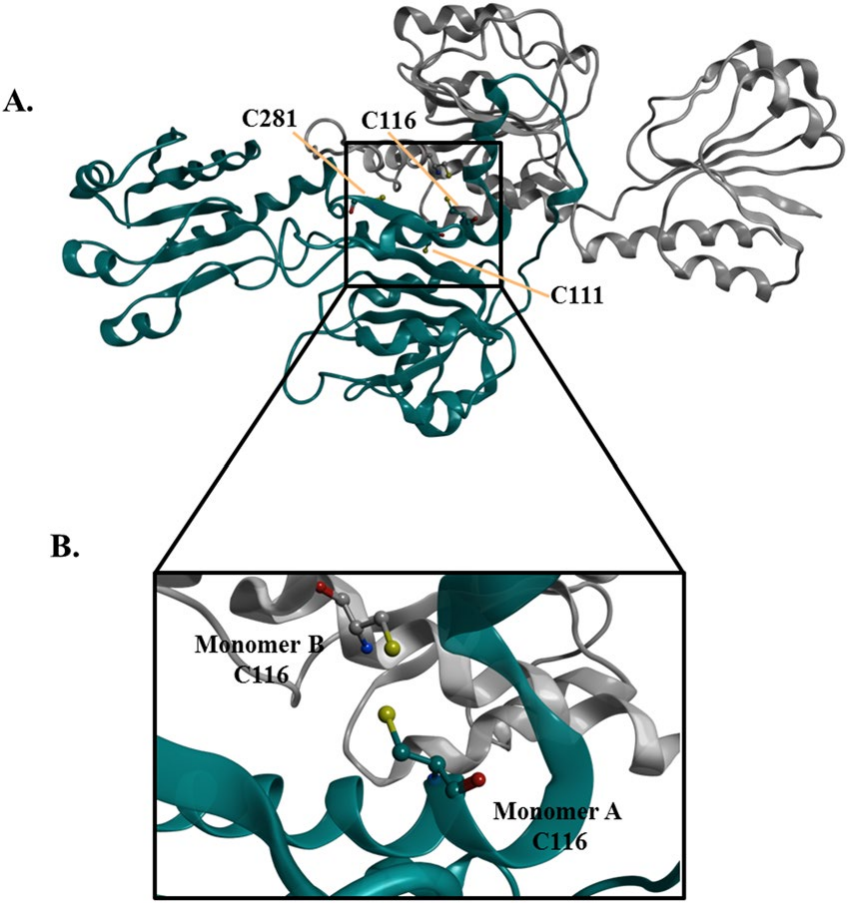
(**A**) Overview of the PHGDH cysteine residues (111, 116 and 281) (PDB code 2G76). (**B**) Zoomed-in region highlighting the targeted Cys116 on the two monomers.

With a view to understand the interaction of DSF with PHGDH on Cys116 at the molecular level, we focused our interest on an already described X-ray crystal structure of a truncated form of PHGDH (Fig. 6). This truncated version shows 11 of the 13 cysteine residues and allowed to visualize the interaction site in more details.

As it can be observed from Fig. 6, the Cys116 residue is located at a key position, at the interface of two PHGDH monomers. According to the results of Marletta^18^, suggesting that S-nitrosation of Cys116 can lead to the formation of a disulfide bridge with the adjacent monomer and then to the formation of an inactive protein, we hypothesized that oxidation of the Cys116 residue by DSF would similarly lead to PHGDH inhibition *via* modification of its oligomeric state.

**Figure 7:**
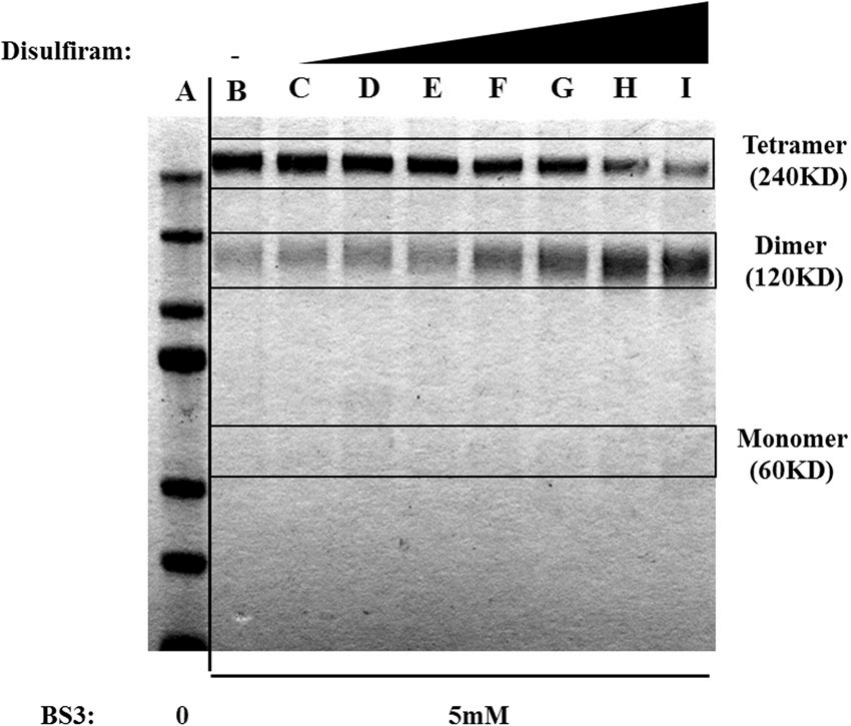
Cross-linking experiment of PHGDH with BS3 at various DSF concentrations **A**. MW marker. **B** 0 µM. **C** 1 µM. **D** 5 µM. **E** 10 µM. **F** 50 µM. **G** 100 µM. **H** 250 µM. **I** 500 µM.). PHGDH was incubated with DSF during 30′ before cross-linking. Lane B was used as control without DSF. Lane A (MW marker) was used to deduce the oligomerization state of PHGDH. Original exposure of the uncropped gel is available in the Supplementary Information File (Fig. S4).

To confirm this hypothesis, a cross-linking experiment, using bis-sulfosuccinimidyl suberate (BS3) as cross-linker, was finally undertaken with PHGDH alone or PHGDH after treatment with increasing concentrations of DSF. As clearly observed from Fig. 7, although PHGDH alone is in a tetrameric form as previously reported^11^, PHGDH inhibition by DSF leads to a concentration-dependent shift from the tetrameric to the dimeric, and to a lesser extent to the monomeric, form of PHGDH, thus corroborating our hypothesis. Since DSF is known to induce the formation of disulfide bridges through the formation of a diethyl(dithiocarbamate) intermediate as exemplified on Fig. 3A ^19^, the results obtained here suggest that DSF would inhibit PHGDH by disruption of the active tetramer either into an inactive dimer resulting from the formation of a disulfide bridge between two Cys116 residue on two adjacent monomers, or to a lesser extent to an inactive diethyl(dithiocarbamate) intermediate monomer.

**Figure 8:**
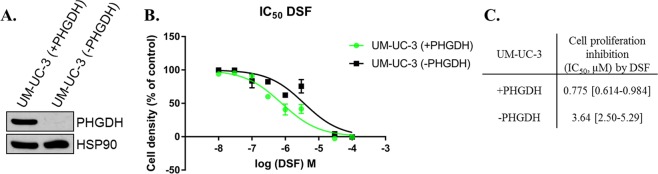
(**A**) Representative immunoblot for PHGDH on UM-UC-3 cancer cells; (**B**) Dose-response curves of DSF. (**C**) UM-UC-3-PHGDH− and UM-UC-3-PHGDH+ cell proliferation inhibition by DSF. All experiments to determine IC50 values were performed in n = 6 at each compound dilution, and all IC50 values were averaged on two or more independent experiments. Under bracket: 95% confidence interval.

Finally to detail the relationship between the ability of DSF to disrupt PHGDH tetramer and its anti-cancer activity, we set out additional experiments aiming to assess the effect of DSF on two cancerous cell lines: UM-UC-3 human transitional cell carcinoma that constitutively express PHGDH (UM-UC-3-PHGDH+) and a variant of these cells that do not express PHGDH (UM-UC-3-PHGDH-) (Fig. 8).

When cells that do not express PHGDH (UM-UC-3-PHGDH-) are treated with DSF, a tumor cell proliferation inhibition (IC_50_) of 3,64 µM is obtained, whereas cells expressing PHGDH (UM-UC-3-PHGDH+) appear to be more susceptible to DSF treatment with an IC_50_ of 0,77 µM. Although the difference remains weak (~5-fold), probably because other anti-proliferative mechanisms are involved upon DSF treatment, tumor cell proliferation inhibition is higher for cells expressing PHGDH.

Our results thus corroborate, at least in cell-based settings, our initial hypothesis that PHGDH inhibition by DSF contributes to its overall anticancer activity.

### Conclusion and Perspectives

In conclusion, in this paper, we have shown that DSF, a FDA-approved aldehyde dehydrogenase inhibitor used as a treatment for chronic alcoholism, and some structural analogues are PHGDH inhibitors. Through biochemical and mass spectrometric experiments, we detailed the mechanism-of-inhibition of PHGDH by DSF and demonstrated that it involves the disruption of the active PHGDH tetramer into either an inactive dimer covalently linked by a disulfide bridge involving Cys116 on adjacent monomers or, to a lesser extent, an inactive monomer intermediate. Using cell-based settings, we also demonstrated our initial hypothesis that PHGDH inhibition by DSF contributes to its overall anticancer activity.

Because it is known that DSF is metabolized *in vivo* (elimination half-life for DSF was reported to be 7.3 h after a single-dose administration of 250 mg of DSF^20^) into notably the diethyldithiocarbamate (DTC) metabolite which undergoes copper complexation, and hence provides anticancer activity for DSF, our results suggest that the non-metabolized circulating DSF could also provide anticancer activity but through a completely different mechanism of action involving PHGDH inhibition. Also, the maximum plasma concentration of DSF (Cmax) being reported to be 1.28 µM, that is around 2-fold higher than the IC_50_ of DSF on PHGDH in the cell-based assay (0.77 µM), one can hypothesized that exposure to DSF *in vivo* could be sufficient to provide anticancer activity *via* PHGDH inhibition.

## Methods

### PHGDH Assay

Enzymatic assay was adapted from a previously described procedure^14^. NADH fluorescence emission (Ex 340 nm/Em 460 nm) was followed over time. Assays were performed in PHGDH assay buffer (50 mM Tris, pH 8.5, and 1 mM EDTA). Substrates and enzyme concentrations were as follows: 3-PG, 240 μM; NAD^+^, 120 μM; glutamate, 30 mM; PHGDH, 12 ng/μL; PSAT1, 20 ng/μL. The final concentration of DMSO in the assay mixture was set to 5%.

### Dilution Experiment

Dilution experiment was conducted following a reported procedure^14^. DSF (5 μM) or DMSO control was incubated with PHGDH for 45 min at 37 °C. Undiluted DSF (5 μM) was included as a positive control for inhibition.

### WT and C116S PHGDH Purification

pET28a human PHGDH and pET28a human C116S PHGDH were transformed into BL21 *Escherichia coli*. A single colony was grown to an OD_600_ 0.6 in 1 L of Luria broth. Protein expression was induced with 1 mM isopropyl thiogalactopyranoside (IPTG). The culture was chilled on ice for 30 min, cultured for 18 h at room temperature and pelleted (6,000 g, 20 min). Pellets were resuspended in 60 mL of lysis buffer (50 mM Tris, pH 8.5, 10 mM MgCl_2_, 300 mM NaCl, 10% glycerol, 5 mM imidazole) and sonicated, and cell debris were pelleted by centrifugation (20,000 × *g*, 30 min). The supernatant was collected and purified using Akta purifier on HisTrap^TM^ FF column (GE Healthcare). After column equilibration with wash buffer (50 mM Tris, pH 8.5, 10 mM MgCl_2_, 300 mM NaCl, 10% glycerol and 30 mM imidazole), bound proteins were eluted with elution buffer (50 mM Tris, pH 8.5, 10 mM MgCl_2_, 250 mM NaCl, 10% glycerol, 250 mM imidazole) and collected (1-mL fractions). Fraction protein content was measured via a Bradford assay. The most concentrated fractions were pooled and dialyzed overnight into 4 L of dialysis buffer (50 mM Tris, pH 8.5, 10 mM MgCl_2_, 250 mM NaCl, 20% glycerol, 0.15% 2-mercaptoethanol). Protein purity was assessed via SDS/PAGE and Coomassie staining.

### Mass spectrometry experiments Orbitrap Lumos

Mass spectrometry experiments were carried out on an Orbitrap Lumos, following a previously reported procedure, with some modifications^21^. A local protein database containing the human PHGDH sequence (accession Uniprot O43175) was used to process the obtained MS/MS data. Mass error was set to 15 ppm for precursor ions and 0.6 Da for fragment ions. Oxidation on Met; disulfiram, (+147.025 Da) and carbamidomethyl (+57.021 Da) were considered as variable modifications on Cys.

### Cross-linking experiments

PHGDH (3 μg) was incubated with DSF (1 µM, 5 µM, 10 µM, 50 μM, 100 μM, 250 µM, 500 μM) or vehicle control (DMSO) in 25 mM HEPES, pH 7.3, and 1 mM NAD^+^ in 18 μL total volume for 30 min on ice. BS3 (S5799; Sigma) cross-linker dissolved in PBS was added to a final concentration of 5 mM and incubated for 30 min under shaking at room temperature. The reaction was then quenched for 15 min by adding 1 M Tris, pH 7.5, to a final concentration of 28 mM. Cross-linked proteins were mixed with sample buffer, boiled for 5 min, and run on SDS/PAGE. Gels were stained with Gelcode blue stain reagent (24592; Thermo) overnight and destained according to the manufacturer’s instructions.

### Cell models and cytotoxicity assay

UM-UC-3 (PHGDH+ and PHGDH-) bladder cancer cells were purchased from the ATCC and cultured in DMEM medium supplemented with 10% heat-inactivated Fetal Bovine Serum (FBS) and 1% penicillin/streptomycin. Cells were seeded at 2500 cells/well in 96-well plates in serine depleted media (MEM). DSF or vehicle (DSMO) was added and cells were grown for 48 hours. Viability was assessed using Presto Blue reagent (Life Technologies) according to manufacturer’s instructions.

### Chemicals

All reagents were purchased from chemical suppliers and used without purification. Copper/DSF complex was obtained according a previously described procedure^22^.

### Immunoblots

Western blot and immunoblot analysis were performed according a reported procedure^23^.

## Supplementary information

Supplementary information
