# Supplementary material for: Anti-alcohol abuse drug disulfiram inhibits human PHGDH via disruption of its active tetrameric form through a specific cysteine oxidation
Source: Sci Rep. 2019 Mar 18;9:4737. doi: 10.1038/s41598-019-41187-0 (PMC6426982; doi:10.1038/s41598-019-41187-0)
Supplement: Supplementary file 1 — Supplementary information [file 41598_2019_41187_MOESM1_ESM.pdf]

Supplementary Info File

Anti-alcohol abuse drug disulfiram inhibits human PHGDH via disruption of its active tetrameric form through a specific cysteine oxidation

Quentin Spillier[1,2], Didier Vertommen[3], Séverine Ravez[4], Romain Marteau[1], Quentin Themans[5], Cyril Corbet[2], Olivier Feron[2], Johan Wouters[5], Raphaël Frédérick[1*]

CONTENT

Triton X effect on DSF IC_{50} S2

DSF inhibition on WT and C116S PHGDH S2

Peptide residues of PHGDH S3

Original Western-blot of the Figure 4 S4

Original exposure for the cross-linking experiment of PHGDH with BS3 S4

Original Immunoblot of the Figure 8 S5

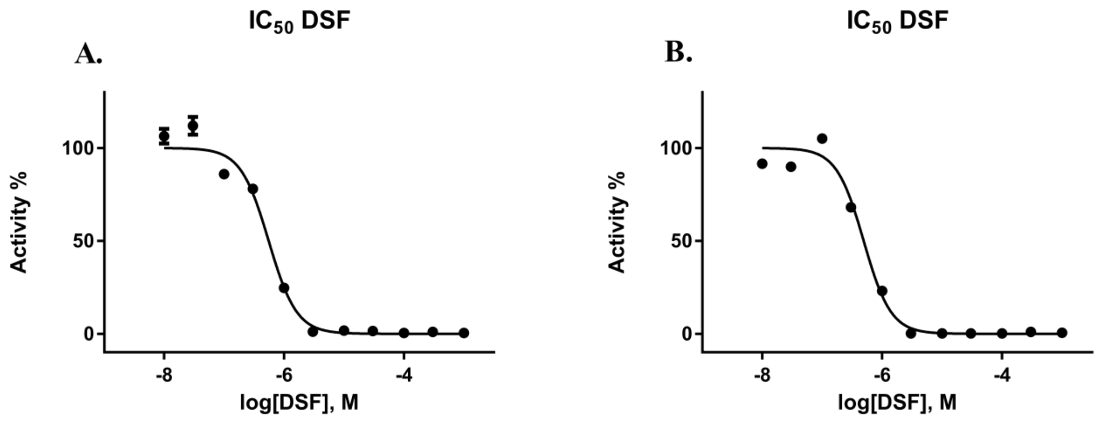

Figure S1. IC_{50} curves of DSF on PHGDH WT **A.** without Triton X (0.53 µM) **B.** with Triton X 0.01% (0.49 µM). All experiments values were performed in triplicates at each compound dilution and error bars show the standard deviation. Data were collected at 37°C with a PHGDH concentration of 12 ng/µL in 50 mM Tris and 1 mM EDTA at pH 8.5.

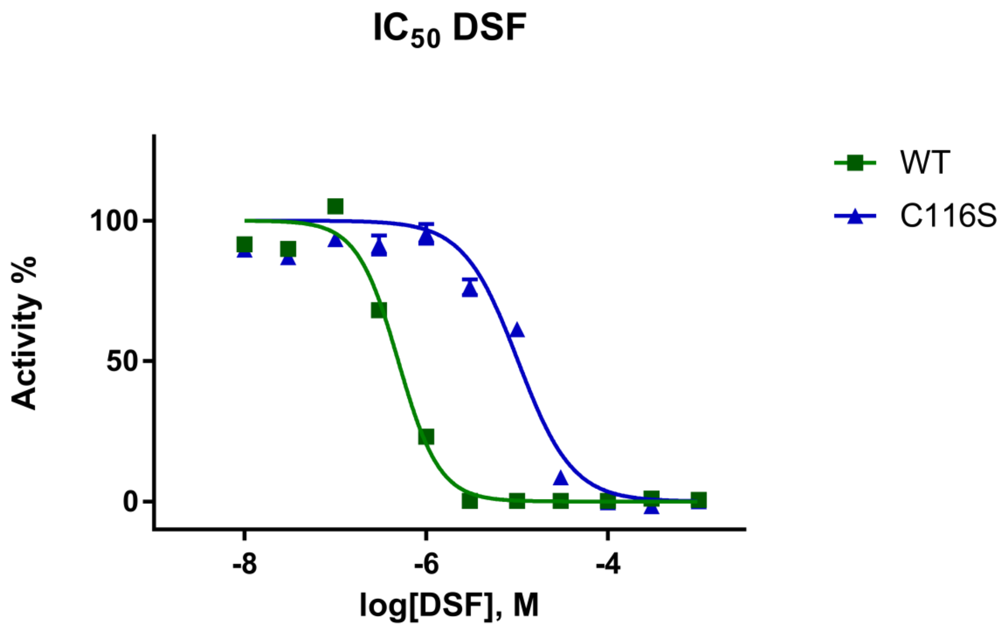

Figure S2. IC_{50} curves of DSF on PHGDH WT (0.49 µM) and C116S (10.23 µM). All experiments values were performed in triplicates at each compound dilution and error bars show the standard deviation. Data were collected at 37°C with a PHGDH concentration of 12 ng/µL in 50 mM Tris and 1 mM EDTA at pH 8.5.

Table S1. PHGDH peptides (after trypsination) showing a disulfiram modification after treatment at **A.** 0.05 µM **B.** 0.5 µM and **C.** 5µM

Peptide sequence[a]	Cys residues	m/z observed MH+ (Da)	m/z calculated MH+ (Da)	Δ (ppm)	Percentage of modified peptides
^{91}KGILVMNTPNGN SLSAAELTCGMIM CLAR^{119}	C111, C116	3155.52390	3155.51082	4.14	C116 = 9.75<br>C111 = 4.87
^{92}GILVMNTPNGNS LSAAELTCGMIMC LAR^{119}	C111, C116	3027.42478	3027.41586	2.95	C116 = 7.69<br>C111 = 1.92

A. DSF concentration 0.05 µM

Peptide sequence[a]	Cys residues	m/z observed MH+ (Da)	m/z calculated MH+ (Da)	Δ (ppm)	Percentage of modified peptides
^{91}KGILVMNTPNGN SLSAAELTCGMIM CLAR^{119}	C111, C116	3171.53720 (Ox M)	3171.50574 (Ox M)	9.92	C116 = 7.14
^{92}GILVMNTPNGNS LSAAELTCGMIMC LAR^{119}	C111, C116	3027.44620	3027.41586	10.02	C116 = 46.67<br>C111 = 6.67

B. DSF concentration 0.5 µM

Peptide sequence[a]	Cys residues	m/z observed MH+ (Da)	m/z calculated MH+ (Da)	Δ (ppm)	Percentage of modified peptides
^{91}KGILVMNTPNGN SLSAAELTCGMIM CLAR^{119}	C111, C116	3155.51438	3155.51082	1.13	C116 = 50.00<br>C111 = 4.16
^{92}GILVMNTPNGNS LSAAELTCGMIMC LAR^{119}	C111, C116	3027.42276	3027.41586	2.28	C116 = 39.65<br>C111 = 10.34
^{271}ALVDHENVISCP HLGASTK^{289}	C281	2137.99712	2138.02649	-13.74	C281 = 1.26

C. DSF concentration 5 µM

[a]Superscripted numbers indicate the amino acid numbering of human PHGDH.

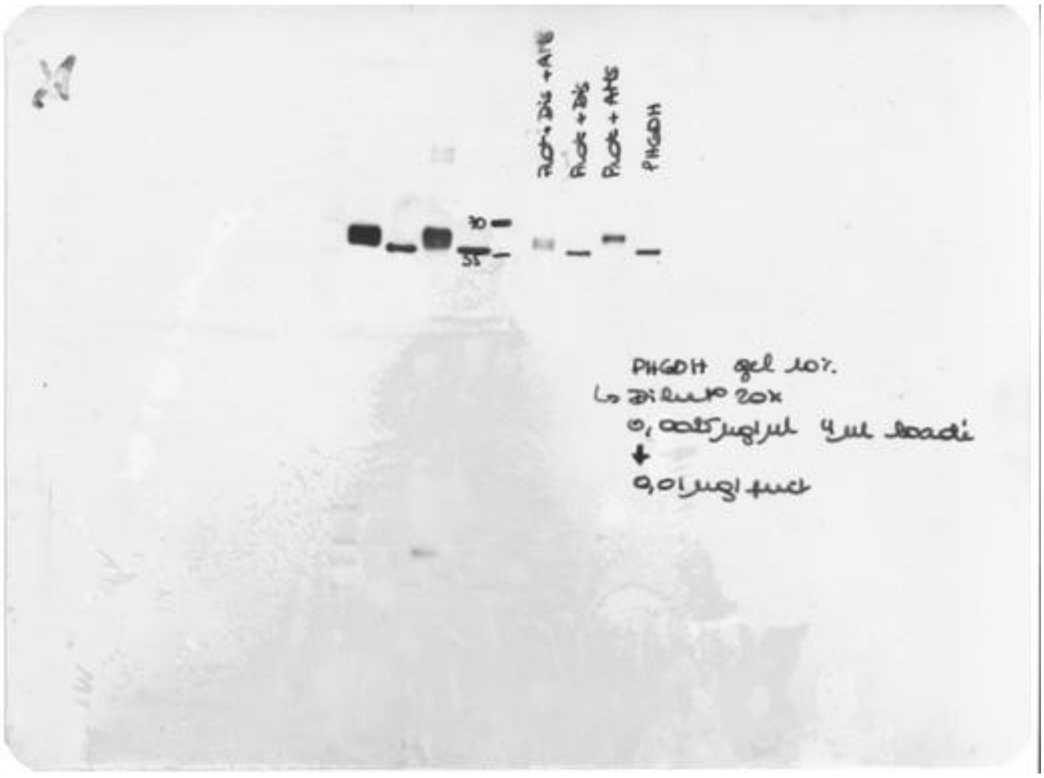

Figure S3. Original Western-blot of the **Figure 4**.

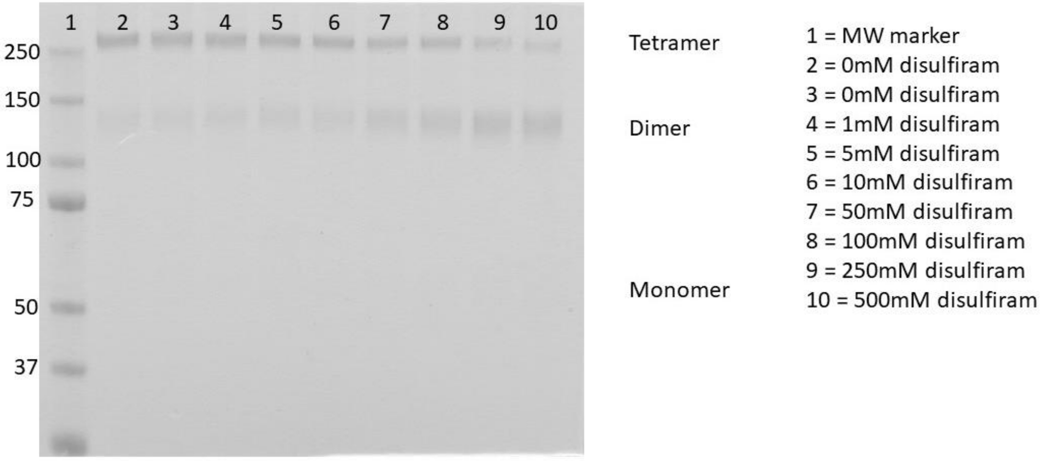

Figure S4. Original exposure for the cross-linking experiment of PHGDH with BS3 at various DSF concentrations. The line 2 (duplication of line 3) was cropped in **Figure 7**.

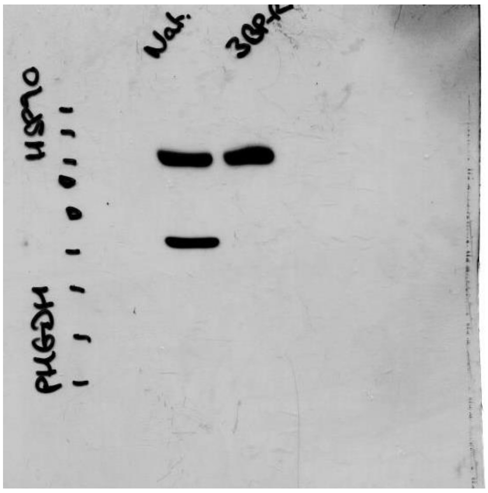

Figure S5. Original Immunoblot of the **Figure 8A**
